# Sustainable synthesis of magnetic petroleum coke/nonanyl chitosan composite for efficient removal of o-nitrophenol

**DOI:** 10.1038/s41598-024-64117-1

**Published:** 2024-06-24

**Authors:** Ahmed M. Omer, Abdelazeem S. Eltaweil, Aly M. Abdelhamed, Eman M. Abd El-Monaem, Gehan M. El-Subruiti

**Affiliations:** 1 Polymer Materials Research Department, Advanced Technology and New Materials Research Institute (ATNMRI), City of Scientific Research and Technological Applications (SRTA-City), P. O. Box: 21934, New Borg El-Arab City, Alexandria Egypt; 2 Department of Engineering, Faculty of Technology and Engineering, University of Technology and Applied Sciences, Ibra, Sultanate of Oman; 3 Chemistry Department, Faculty of Science, Alexandria University, Alexandria, Egypt; 4 Environmental department, EPROM-MIDOR Refinery, P. O. Box: 1001, Alexandria, Egypt

**Keywords:** Nonanyl chitosan, Petroleum coke, Nitrophenol, Adsorption, Kinetics, Environmental chemistry, Materials science, Polymer chemistry

## Abstract

Worldwide industrialization has grown at a rapid pace, contaminating water resources, particularly with phenolic pollutants that pose a risk to aquatic systems and human health. The goal of this study is to create an inexpensive magnetic composite that can effectively remove nitrophenol (o-NP) using adsorptive means. In this instance, a nonanyl chitosan (N-Cs) derivative was synthesized and then combined with activated petroleum coke (AP-coke) and magnetic Fe_3_O_4_ to boost its adsorbability towards o-NP and to facilitate its separation. Fourier transform infrared spectroscopy (FTIR), scanning electron microscopy (SEM), X-ray diffractometer (XRD), Vibrating sample magnetometer (VSM), X-ray photoelectron spectroscopy (XPS), and zeta potential were employed to characterize the magnetic composite. The experimental results indicated that the Fe_3_O_4_/AP-coke/N-Cs composite possesses a greater affinity toward o-NP with a maximal efficiency reached 88% compared to 22.8, 31.2, and 45.8% for Fe_3_O_4_, AP-coke and N-Cs, respectively. The equilibrium adsorption data coincided with the Langmuir, Freundlich, and Temkin isotherm models, with a maximum adsorption capacity of 291.55 mg/g at pH 6, whereas the pseudo second order kinetic model offered the best fit to the experimental data. Besides, the developed adsorbent preserved satisfactory adsorption characteristics after reuse for five successive cycles. The proposed adsorption mechanism involves the H-bonding, π-π interaction, hydrophobic interactions and electron donor-acceptor interactions. These findings hypothesize that the constructed magnetic composite could efficiently remove nitrophenols from polluted water with high performance and ease-separation.

## Introduction

Recently, scientific researchers have shown considerable anxiousness regarding the effects of phenolic chemicals on human life and the aquatic environment^1^. Petroleum refineries, petrochemical, coke oven plants, steel mills, synthetic resins, pharmaceuticals, coal gas, paints, mine discharge and plywood industries are considered the major sources of phenolic waste^2,3^. A possible cause of their existence in the water is the breakdown of naturally occurring organic compounds brought about by internal and industrial waste disposal as well as overflows from agricultural areas. Under physical, chemical, and biological interactions, these phenolic compounds have the tendency to change into other, more hazardous moieties than their original ones^4,5^.

Particularly, when phenol and nitrite ions mix in water, o-nitrophenol and p-nitrophenol are created, since the reaction happens across a broad pH range and is affected by UV radiation (sunlight)^2,4,6^. Specifically, o-Nitrophenol (o-NP or 2-NP) is a chemical compound with notable implications for both aquatic ecosystems and human well-being (Abd El-Monaem et al.^28^). Within aquatic habitats, o-NP presents a hazard to the intricate balance of ecosystems, proving toxic to diverse organisms such as fish, invertebrates, and algae. Its introduction can disrupt ecosystem processes, resulting in diminished biodiversity and compromised nutrient cycles (Ma et al.^6^). Regarding human health, exposure to o-NP may arise through ingestion, inhalation, or skin contact, eliciting immediate symptoms such as skin, ocular, and respiratory irritation^7^. Prolonged exposure to o-NP is also linked to potential carcinogenic effects, with research indicating an elevated likelihood of specific cancers such as bladder cancer. Besides, o-NP falls under the classification of endocrine-disrupting chemicals (EDCs), capable of perturbing hormonal systems and fostering conditions conducive to reproductive and developmental disorders^8^.

As a result, USEPA identified 2,4-nitrophenol (2,4-DNP), 4-nitrophenol (4-NP), and 2-nitrophenol (2-NP) as primary contaminants with an MCL < 10 ng L^–1^^9^. Therefore, techniques that are both economical and efficient are required to rid water bodies of these harmful substances. To remove nitrophenols, a number of approaches have been used, including the Fenton process, catalytic reduction, acoustic cavitation, catalytic ozonation, adsorption, biological treatment with or without oxygen, and acoustic catalytic breakdown^10–15^. Due to its low cost, easy processing, great performance, low energy consumption, and ability to produce treated wastewater of the highest quality on a small- and large-scale without secondary pollution, adsorption has grown in popularity among environmentalists^16,17^. Many adsorbent materials have been used as effective adsorbents, including silica, carbon-based materials, natural clays, MOFs materials, organic wastes, and polysaccharides^18–21^.

Because polysaccharide-based adsorbents are abundant, environmentally friendly, biodegradable, non-toxic, and have the ability to interact chemically and physically with a wide range of molecules, they have garnered significant interest in recent decades for the purposes of extracting, purifying, and separating chemicals from water^22,23^. Chitosan (Cs) is a kind of polysaccharide created by simple deacetylation of chitin, which considered the main constituent of the exoskeleton of crab shells^24,25^. The existence of reactive hydroxyl and amine groups along the chitosan backbone allows various physical and chemical modification processes such as Schiff base formation, crosslinking, grafting and composite formation for widen its application range^26–29^. Owing to its excellent advantages including low-cost production, eco-friendly, non-toxicity, ease of modification and biodegradability chitosan has been employed as potential candidate in countless fields such as industrial, water treatment, pharmaceutical and medical fields^30–32^. Also, chitosan has been effectively used as adsorbent material for adsorptive removal of various noxious contaminates such as organic dyes, pharmaceutical residues and heavy metals^33^. Nevertheless, chitosan suffers from its limited surface area, weak mechanical properties and little adsorbability towards organic pollutants resultant from its higher hydrophilicity^34^.

Petroleum coke (AP-coke) is a hydrophobic, carbonaceous, black solid material that mostly look like coal, while a tiny portion comprises carbonaceous fibers. PC is typically made up of carbon^31,35^, accounting for 90% of its mass, with the residual being mainly made up of hydrogen, nitrogen, oxygen, sulphur. Characteristically, the classification of the source of oil may be done using the petcoke’s comparatively high amounts of silicon, trace metals. It has vast proficiency to prepare various kinds of carbon-based materials like activated carbon^36^, carbon nanotubes. Activated carbon can be generated from PC, effectively applied for the removal of tetracycline^10^, phenols^11,37^, organic acids. Moreover^12^, PC is a non-porous (surface area <10 m^2^/g) solid containing ahigh content of numerous impurities, such as metal compounds, sulfur (1–8%). Therefore, the modification of petroleum coke is essential for developing its pore structure, surface area, as well as removing the impurities.

Our study intended to fabricate a developed magnetic Fe_3_O_4_/AP-coke/N-Cs adsorbent, exploiting the remarkable features of Fe_3_O_4_, AP-coke, and nonanyl chitosan (N-Cs) for removing o-NP from wastewater. Notably, the excellent hydrophobic character of N-Cs strengthens the hydrophobic interactions between Fe_3_O_4_/AP-coke/N-Cs and o-NP. Furthermore, AP-coke boosts the adsorbability of Fe_3_O_4_/AP-coke/N-Cs toward the o-NP molecules, and Fe_3_O_4_ could increase the removal aptitude of the composite and facilitate its separation via a magnet. The magnetic composite was characterized by several analytical tools: FTIR, XPS, SEM, zeta potential, and XRD. Furthermore, parameters affecting the adsorption process were investigated and optimized, such as contact time, o-NP concentration, adsorbent dosage, pH medium, and temperature. Various kinetics and isotherms models were applied to define the interaction type between Fe_3_O_4_/AP-coke/N-Cs and o-NP. In addition, the adsorption mechanism was proposed according to XPS spectra of authentic and used Fe_3_O_4_/AP-coke/N-Cs composites.

Thanks to the remarkable features of the magnetic Fe_3_O_4_/AP-coke/N-Cs adsorbent that enables it to extend out of bench scale to industrial scale, comprising abundant and costless components, facile processing without using complicated instruments or consuming high energy, excellent recyclability, fast and easy separation from the adsorption medium, and promising adsorbability.

## Experimental

### Materials

Chitosan (M.wt. 100000–300000D, DD=95%) was purchased from Sigma Aldrich Co. (Germany). Glacial acetic acid (assay 99%, Ethanol (assay 99%), Sodium hydroxide pellets (purity 98%) and Nitric acid (69%) were purchased from Aladdin Reagent Co., Ltd. (China). Ferric chloride hexahydrate (FeCl_3_.6H_2_O; purity 97%) and ferrous (II) Sulfate Heptahydrate (FeSO_4_·7H_2_O; purity 99%) were supplied by Loba Chemie (India). Petroleum coke was obtained from Middle East Oil Refinery (MIDOR) Egypt.

### Activation of petroleum coke (AP-coke)

The activation of petroleum coke (P-coke) was achieved by co-activation process^38^. Accurate 10 g of P-coke was added to nitric acid solution (10.7 N) and stirred for 24 h at room temperature. Next, the mixture was filtered and the resultant powder was subsequently transferred into a crucible and subjected to physical activation by leaving it in a muffle furnace at 650 ºC for 3 h. The activated P-cock was taken out and, cooled in a dry atmosphere free of moisture and washed a couple of times before drying at 80ºC overnight.

### Preparation of nonanyl chitosan Schiff base (N-Cs)

Nonanyl chitosan Schiff base derivative was prepared based on the authors previous work^39^. In brief, chitosan was dissolved in 50 mL of acetic acid solution (2% w/v) for 6 h at room temperature to have final concentration of 2% w/v. An exact quantity of nonanal (1.86 mM) was dissolved in ethanol in (10 mL) and dropped leisurely in chitosan solution under incessant stirring at 50 °C for additional 6 h. The resultant product exhibited an intense yellow color as a result of nonanyl chitosan Schiff base (N-Cs) formation. Afterwards, N-Cs solution was precipitated using sodium hydroxide (5% w/v), accompanied with filtration. The produced N-Cs Schiff base was washed with a mixture of distilled water and ethanol and left overnight for drying in a vacuum oven at 60 °C.

### Preparation of Fe_3_O_4_/AP-coke/N-Cs magnetic composite

**Scheme 1:**
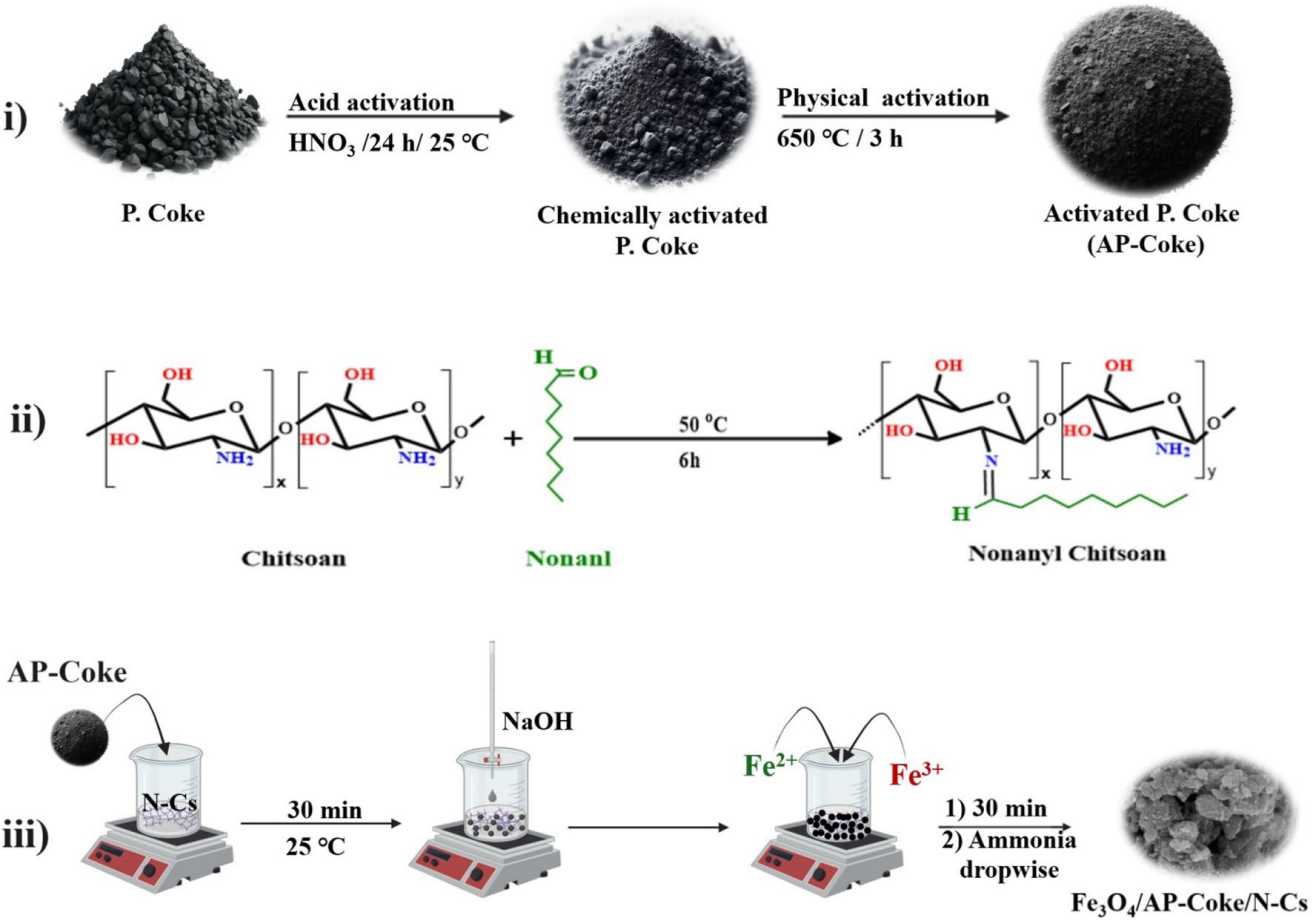
A schematic pathway for the synthesis of Fe_3_O_4_/AP-coke/N-Cs magnetic composite.

A precise quantity of the synthetized N-Cs Schiff base derivative (1.5 g) was dissolved in 50 mL of demineralized water/ethanol (2.5:1) and 5 % (w/w) aqueous solution of acetic acid and stirred for 3 h until the mixture became homogenous. Then, 1.5 g of AP-coke were added to the N-Cs solution and left under stirring at room temperature for 30 min. Subsequently, 10 mL of NaOH solution (5 mmol/L) was added drop-by-drop to precipitate AP-coke/N-Cs mixture^40^. Hence, NaOH, as a strong base, is able to increase the pH of the mixture and can consequently react with the acidic deprotonated functional groups present in chitosan, such as amino and hydroxyl groups. This pH-driven change in solubility can trigger the precipitation of the AP-coke/N-Cs composite. Later, 0.5 g of AP-coke/N-Cs composite was dispersed in 23 mL of demineralized water and afterward heated to 70 °C. An amount of 0.58 g FeCl_3_.6H_2_O and 0.3 g FeSO_4_.7H_2_O were dissolved in 40 mL of demineralized H_2_O and followed with addition to the composite mixture. The mixture was stirred for 30 min and then, 30 mL of ammonia was added dropwise to obtain Fe_3_O_4_/AP-coke/N-Cs composite. The formed magnetic composite was washed with demineralized water and then overnight dried in an oven at 100°C. A schematic diagram describes the synthesis of Fe_3_O_4_/AP-coke/N-Cs magnetic composite is presented in Scheme 1.

### Adsorbent characterization

The chemical structure of the Fe_3_O_4_/AP-coke/N-Cs composite was characterized by Fourier Transform Infrared Spectrometer (FTIR- Shimadzu8400S). The morphological properties were examined by a Scanning Electron Microscopy (SEM- S4800, Hitachi, Japan). X-ray Diffractometer (XRD- MAC Science M03XHF) was employed to investigate the crystallinity of the developed composite, while the magnetic properties were examined by Vibrating sample magnetometer (VSM), and Zeta potential analyzer (ZP- Malvern, UK).Further, the elemental-surface composition was inspected by X-ray Photoelectron Spectroscopy (XPS, Axis Ultra DLD, Shimadzu, Japan).

### Batch adsorption studies

The influences of controlling adsorption factors on the adsorption aptitude of o-NP onto the surface of Fe_3_O_4_/AP-coke/N-Cs composite were studied using a batch adsorption mode as follows: (i) The concentration of o-NP ranged between 50 and 200 mg/L during a contact time extended to 140 min. (ii) The adsorbent dosage was ranged from 0.005 to 0.02 g. (iii) The pH of the adsorption medium was studied in the range of 2–12. (iv) The adsorption temperature was changed at the temperature range of 25–45 °C. The adsorption process was performed in a shaking water bath at a constant speed of 100 rpm. After an adsorption time, an o-NP sample was withdrawn, and then the o-NP concentration was analyzed using a UV-vis spectrophotometer at λ_max_ of 345 nm. The removal efficiency (R %) and adsorption capacity (q) of o-NP onto Fe_3_O_4_/AP-coke/N-Cs were estimated from Eqs. (1) and (2)^41^.

1 $$\text{q }=\frac{({C}_{0}-{C}_{t})V}{m},$$

2 $$\text{R }\left({\%}\right)=\frac{({C}_{0}-{C}_{t})}{{C}_{0}} \times 100$$

where, C_0_ and C_t_ symbolize the initial o-NP concentration and its concentration at an interval time, respectively. m and V represent the quantity of used adsorbent composite and the phenol solution volume, respectively.

## Results and discussion

### Characterization

#### FTIR spectra

**Figure 1:**
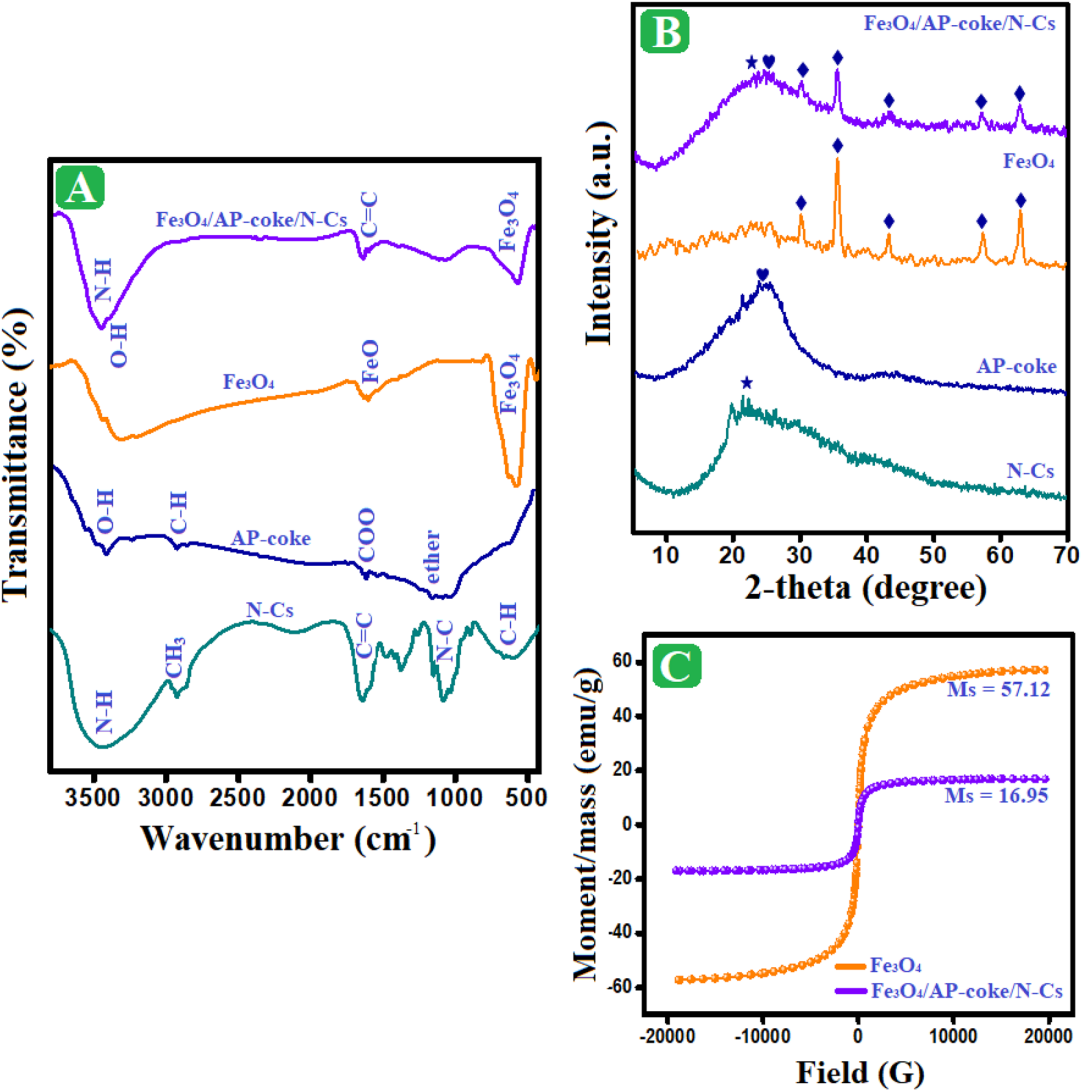
(**A**) FTIR, (**B**) XRD of N-Cs, AP-coke, Fe_3_O_4_, and Fe_3_O_4_/AP-coke/ N-Cs composite, and (**C**) VSM of Fe_3_O_4_ and Fe_3_O_4_/AP-coke/N-Cs composite.

The FTIR bands of N-Cs, AP-coke, Fe_3_O_4_, and Fe_3_O_4_/AP-coke/N-Cs composite are exhibited in Fig. 1A. The N-Cs spectrum revealed the characteristic peaks of C–H wagging at wavenumbers 663 and 895 cm^–1^. The bands at 1033, 1258, and 1420 cm^–1^ are allocated to C–O–C, N–C, and C–OH bonds, respectively. The belonging bands to the wagging vibration of CH_2_ manifested at 1376 cm^–1^, while the band at 2922 cm^–1^ is attributed to the CH_3_ vibration. In addition, the existing band at 1151 cm^–1^ corresponds to C–H out of the plane deformation^42^. The appeared bands at 1471 and 3375 cm^–1^ are ascribed to N–H, and the band at 1640 cm^–1^ is assigned to C=C. Thus, the FTIR spectrum of N-Cs could confirm the functionalization of Cs by nonyl group^43,44^. The AP-coke spectrum elucidated bands at 1043 and 3413 cm^–1^, which are accompanied by ether and hydroxyl, subsequently. In addition to the bands at 1615 and 2925 cm^–1^ related to COO and aliphatic C–H, respectively^45^. The Fe_3_O_4_ spectrum illustrated its distinguishing band at 576 cm^–1^, and the bands at 626 and 1402 cm^–1^ corresponded to Fe-O stretching vibration. The bands at 1630 and 820 cm^–1^ belong to OH bending and OH stretching vibration band manifested at 3305 cm^–1^^12^. Finally, the spectrum Fe_3_O_4_/AP-coke/N-Cs could infer the homogeneity between Fe_3_O_4_, N-Cs, and AP-coke, where their distinct bands appear with lower intensity compared to the authentic components.

#### XRD patterns

The crystallographic patterns of N-Cs, AP-coke, Fe_3_O_4_, and Fe_3_O_4_/AP-coke/N-Cs composite are illustrated in Fig. 1B. The N-Cs pattern denoted the amorphous character of N-Cs with a wide band at 21.8º. Similarly, the amorphous nature of AP-coke was evinced via its crystallographic pattern, where broadband appeared at 24.5º. The Fe_3_O_4_ pattern depicts the distinguishing peaks of magnetite at 30.2º, 35.7º, 43.3º, 57.04º, and 62.7º, following 220, 311, 400, 511, and 440 planes, respectively^46^. The Fe_3_O_4_/AP-coke/N-Cs pattern implied the well-fabrication of the composite since the characteristic peaks of magnetite manifested obviously. In addition, the appearance of overlapping of the AP-coke and N-Cs peaks forms a broadband centered at 24.1º. This finding was consistent with the FTIR results that suggested a good combination between Fe_3_O_4_, AP-coke, and N-Cs.

#### VSM hysteresis loop

Figure 1C represents the hysteresis loops of Fe_3_O_4_ and Fe_3_O_4_/AP-coke/N-Cs composite. The magnetism measurements clarified the superparamagnetic property of Fe_3_O_4_ in which the coercivity was 8.61 G. While the coercivity of Fe_3_O_4_/AP-coke/N-Cs composite increased to 31.73 G, reflecting the ferromagnetic character of the composite. Furthermore, the saturation magnetization of Fe_3_O_4_ and Fe_3_O_4_/AP-coke/N-Cs composite were 57.12 and 16.95 emu/g, implying a decline in the magnetism of Fe_3_O_4_ after blinding with AP-coke and N-Cs. This decline in the Fe_3_O_4_ magnetism is due to the non-magnetic performance of AP-coke and N-Cs and the low proportion of Fe_3_O_4_ in the composite^47^.

#### Zeta potential

The net charges magnitude on the Fe_3_O_4_/AP-coke/N-Cs composite was measured by zeta potential, as elucidated in Fig. S1. It was found that the point of zero charges was 4.67, reflecting the Zwitterionic-nature of Fe_3_O_4_/AP-coke/N-Cs around pH = 4.67. The composite has positive charges in a highly acidic medium and negative charges in alkaline media. Hence, Fe_3_O_4_/AP-coke/N-Cs could adsorb both anionic and cationic contaminants from wastewater.

#### XPS spectra

**Figure 2:**
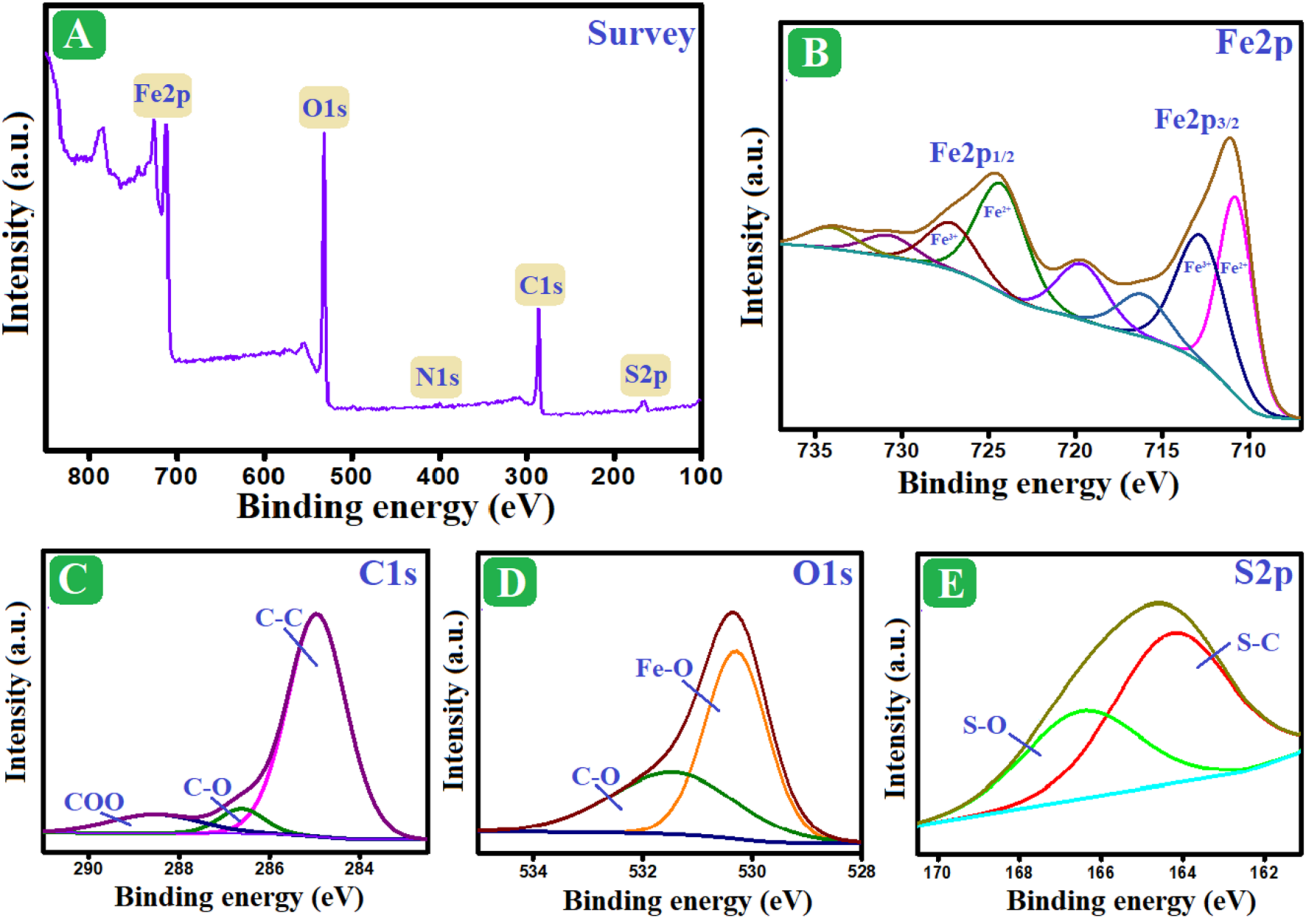
XPS spectra of Fe_3_O_4_/AP-coke/N-Cs composite; (**A**) Wide-spectrum, (**B**) Fe2p, (**C**) C1s, (**D**) O1s, and (**E**) S2p.

The elemental and chemical composition of the Fe_3_O_4_/AP-coke/N-Cs composite was investigated using XPS analysis (Fig. 2A–E). The wide-spectrum signalized that Fe_3_O_4_/AP-coke/N-Cs is composed of sulfur (S2p), nitrogen (N1s), iron (Fe2p), carbon (C1s), and oxygen (O1s). The iron spectrum clarified the presence of ferrous species, where their distinctive peaks manifested at 710.75 and 724.27 eV; in addition, the existing ferric species at 712.74 and 727.13 eV^48^. The carbon spectrum illustrated the corresponding peaks to C–O, C–C, and COO at 286.62, 284.96, and 288.53 eV, respectively. The oxygen spectrum depicted the peaks at 530.3 and 531.44 eV, which are allocated to Fe–O and C–O, respectively. The sulfur spectrum denoted the belonging peak to S–C at 164.25 eV and S–O at 166.49 eV. The nitrogen-spectrum (Fig. S2) clarified the N-H peak at 400.03 eV. Consequently, the XPS spectra denoted the well-combination between Fe_3_O_4_, AP-coke, and N-Cs since the belonging peaks to their chemical composition appeared.

#### SEM

**Figure 3:**
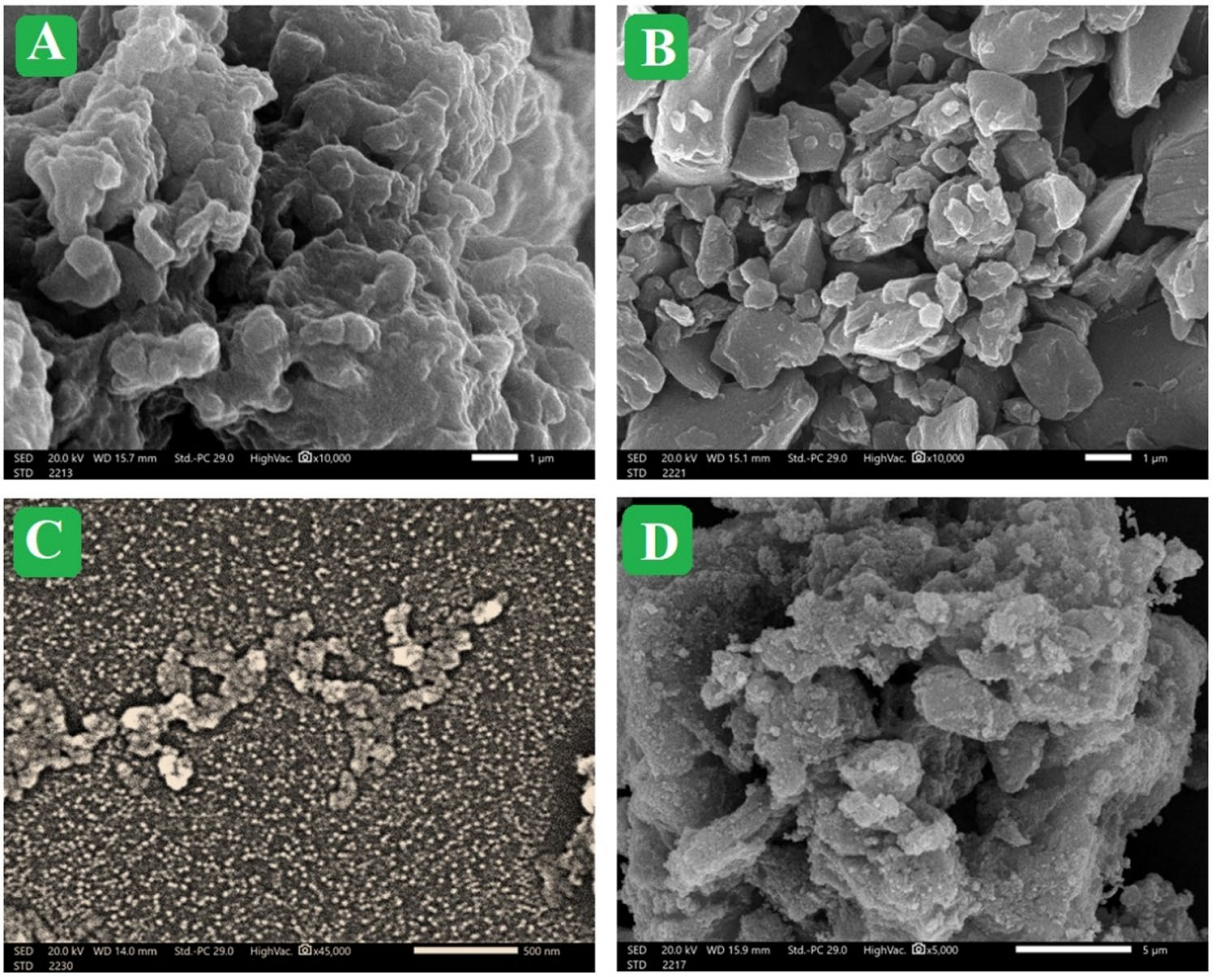
SEM images of (**A**) N-Cs, (**B**) AP-coke, (**C**) Fe_3_O_4_, and (**D**) Fe_3_O_4_/AP-coke/N-Cs composite.

The SEM images of N-Cs, AP-coke, Fe_3_O_4_, and Fe_3_O_4_/AP-coke/N-Cs composite were exhibited in Fig. 3A–D. By analyzing N-Cs under the SEM, it was observed that it has a sponge-like morphology with a wrinkled and rough outer surface. The specifications of the N-Cs surface enable it to carry the particles of Fe_3_O_4_ and AP-coke. The AP-coke morphology looks like irregular rocky shapes with quite uneven sizes. The Fe_3_O_4_ image elucidated that its particles are almost spheroidal, with a tiny size on a nano-scale. The Fe_3_O_4_/AP-coke/N-Cs image implied spreading the Fe_3_O_4_ and AP-coke particles onto the N-Cs surface, assuring the feature of the N-Cs surface to act as a supporter.

### Optimization of the adsorption process

#### Impact of adsorbent compositions

**Figure 4:**
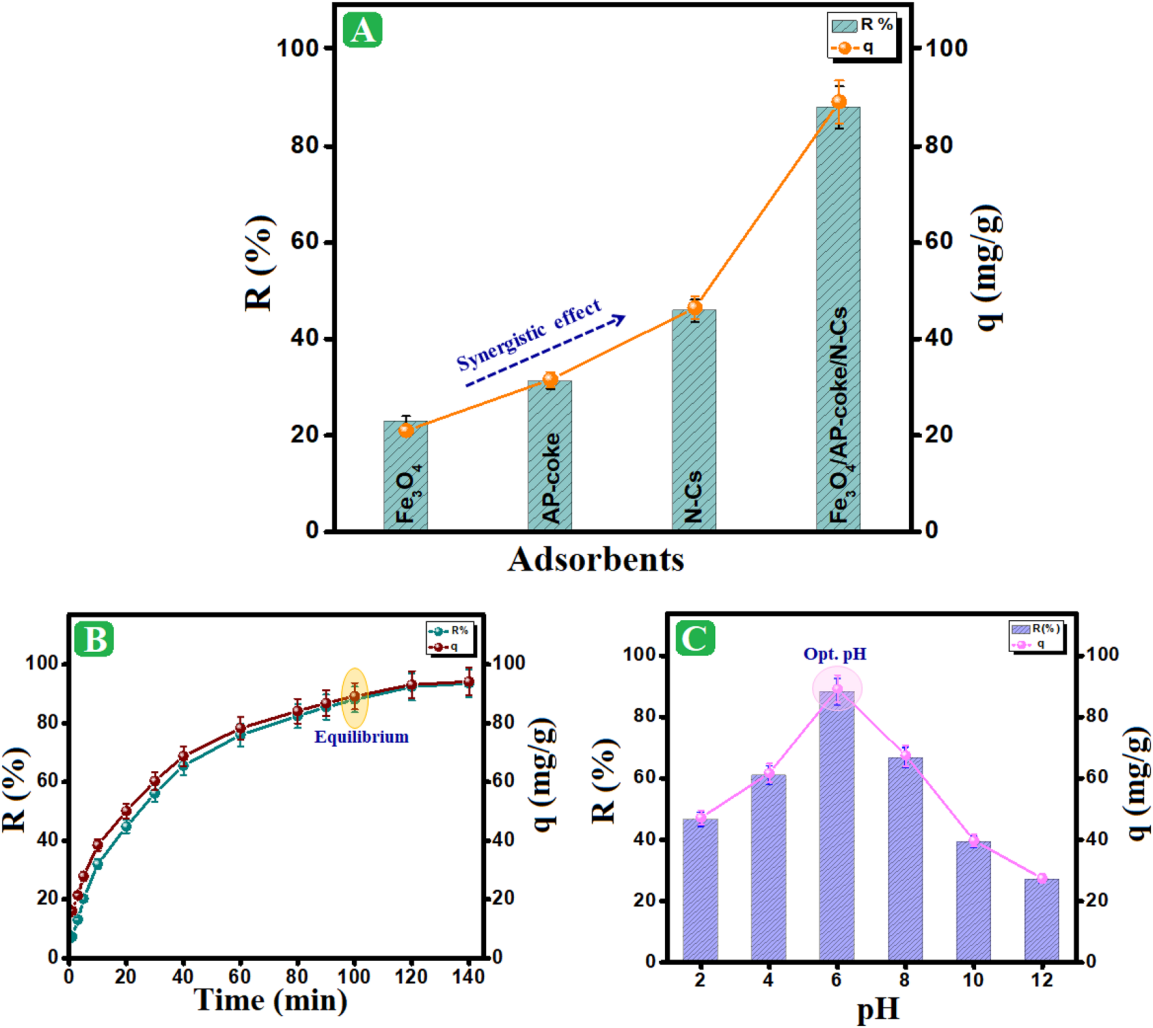
(**A**) Comparison test between capabilities of Fe_3_O_4_, AP-coke, N-Cs, and Fe_3_O_4_/AP-coke/N-Cs composite towards the adsorption of o-NP, (**B**) Effect of contact time, and (**C**) Effect of pH medium on the o-NP adsorption aptitude.

The adsorbability of the composite and its components towards o-NP was evaluated at the same adsorption parameters [pH = 6, C_o_ of o-NP = 50 mg/L, temperature = 25 ºC, dose of adsorbent = 0.01g, and Time = 100 min], as shown in Fig. 4A. The adsorption capacity of Fe_3_O_4_, AP-coke, and N-Cs were 21, 31.6, and their removal (%) were 46.47, 22.85, 31.22, and 45.89 %, respectively. On the other hand, the adsorption capability of the magnetic Fe_3_O_4_/AP-coke/N-Cs composite was remarkable compared to the authentic component since its adsorption capacity and removal (%) were 89.2 mg/g and 88.1 %, sequentially. This outstanding performance of Fe_3_O_4_/AP-coke/N-Cs owes to the implication numerous of interactions between o-NP molecules and the composite, including H-bonding, π-π interaction, hydrophobic interactions, and electron donner/acceptor interaction that could be provided by Fe_3_O_4_, AP-coke, and N-Cs.

#### Impact of contact time

The impact of contact time on the adsorption of o-NP on the surface of Fe_3_O_4_/AP-coke/N-Cs magnetic composite was illustrated in Fig. 4B. The findings demonstrated that the adsorption process was quickly enhanced over the first 60 min with a maximum adsorption capacity of 78.30 mg/g and a maximum removal (%) of 76 %. Then, the adsorption process took place in a slow rate until it reached equilibrium in 100 min, with a maximum adsorption capacity and removal (%) values of 89.2 mg/g and 88.07 %, respectively. This phenomenon can be explained by an increase in the number of o-NP molecules that diffuse throughout the film fluid around the sufficient free adsorption sites of the adsorbent surface during the initial adsorption stage^49^. Nevertheless, there was no noticeable effect on the adsorption performance with increasing the contact time beyond 100 min, since most of adsorption sites were saturated by o-NP molecules.

#### Impact of pH

The most important variable influencing the adsorption process is unquestionably pH. Thus, as shown in Fig. 4C, its impact on the adsorption of o-NP by Fe_3_O_4_/AP-coke/N-Cs magnetic composite was investigated at a pH range of 2–12. At pH 6, the o-NP adsorption onto Fe_3_O_4_/AP-coke/N-Cs composite demonstrates an excellent adsorption performance, as demonstrated by the highest adsorption capacities of 89.2 mg/g and 88.07 %, respectively. This finding may be explained by the fact that o-NP has a pKa of 7.23, indicating that it exists in the molecule form at acidic circumstances^6,50^. Therefore, certain chemical and physical interactions, such as the π-π interaction and H-bonding, play a more important role in the adsorption mechanism of o-NP than the electrostatic interaction. On the other hand, as pH rose beyond 6, there was a noticeable decline in adsorption performance with increasing pH to 12 as recorded by the lowest values of 27.4 mg/g and 26.05 % for both the adsorption capacity and the removal (%) of o-NP. The strong repulsion forces between the negatively charged magnetic composite and the anionic o-NP at higher pHs may be responsible for those results.

#### Impact of o-NP concentration

**Figure 5:**
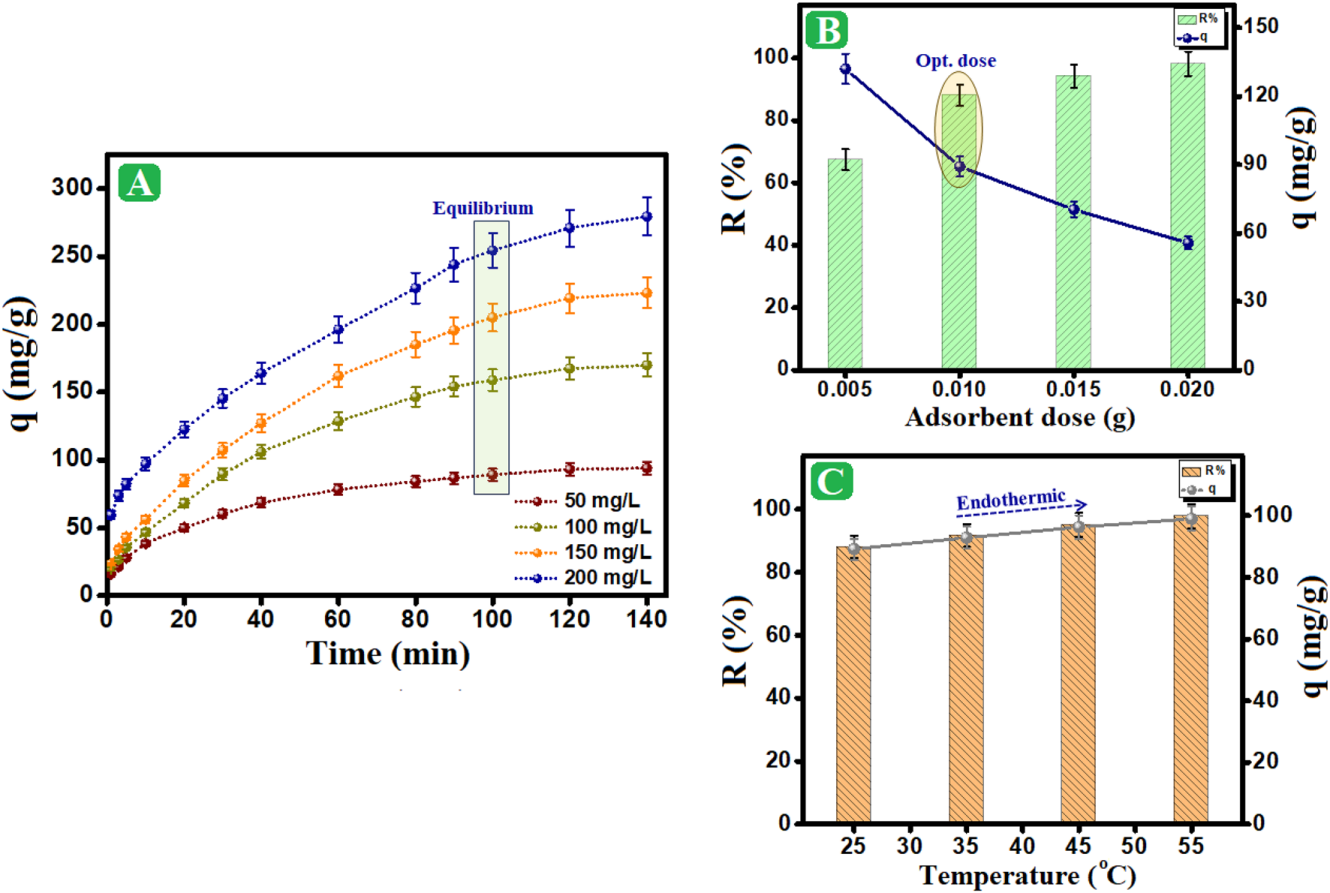
Impact of (**A**) Initial concentration of o-NP, (**B**) Adsorbent dose, (**C**) and Adsorption temperature on the adsorption of o-NP onto Fe_3_O_4_/AP-coke/N-Cs magnetic composite.

Figure 5A shows the impact of the initial concentration of o-NP on the adsorption capacity and removal (%) values. The results refereed that the adsorption capacity meaningfully increased from 89.2 to 254.6 mg/g with increasing the o-NP concentration from 50 to 200 mg/L. Indeed, the increase in the initial concentration of pollutant reinforces its driving force that overwhelms the mass transfer struggle and enhances the migration from the bulk solution to the surface of the adsorbent. Nevertheless, the removal (%) value was declined from 88.1 to 52.8 % as the concentration of o-NP solution increased from 50 to 200 mg/L, which most probable due to the increase in the driving force reduction of the free adsorption sites, in which the entire surface sites on the surface of Fe_3_O_4_/AP-coke/N-Cs had saturated using a certain o-NP concentration.

#### Impact of adsorbent dose

Figure 5B illustrates how the adsorption performance of o-NP is affected by an increase in the dose of the adsorbent. As anticipated, the removal percentage of o-NP increases from 67.4 to 98.2 % with the increase in the adsorbent dose from 0.005 to 0.02 g. The observed result may be attributed to the overabundance of adsorption active sites on the Fe_3_O_4_/AP-coke/N-Cs magnetic composite surface, which increases with increasing dose and subsequently enhances the removal percentage (%) of o-NP. On contrary, as the dose was increased, the adsorption capacity value decreased from 132 to 55.7 mg/g. This may be explained by increasing the number of unsaturated active sites with raising the Fe_3_O_4_/AP-coke/N-Cs dosage at a constant o-NP concentration^51,52^.

#### Impact of temperature

As seen in Figure 5C, the impact of adsorption medium temperature on the adsorption process was examined in the 25–45 °C range. The findings showed that when the temperature was raised between the range of 25–45 °C, the adsorption capacity and the removal percentage both marginally rose, going from 89.2 mg/g and 88% to 99 mg/g and 97.2%, respectively. These results could be explained by increasing the segmental motion of the adsorbent composite with rising temperature. This would increase the pace at which o-NP disperses over the adsorbent's exterior boundary layer, boosting the adsorption profile.

### Kinetic study

**Figure 6:**
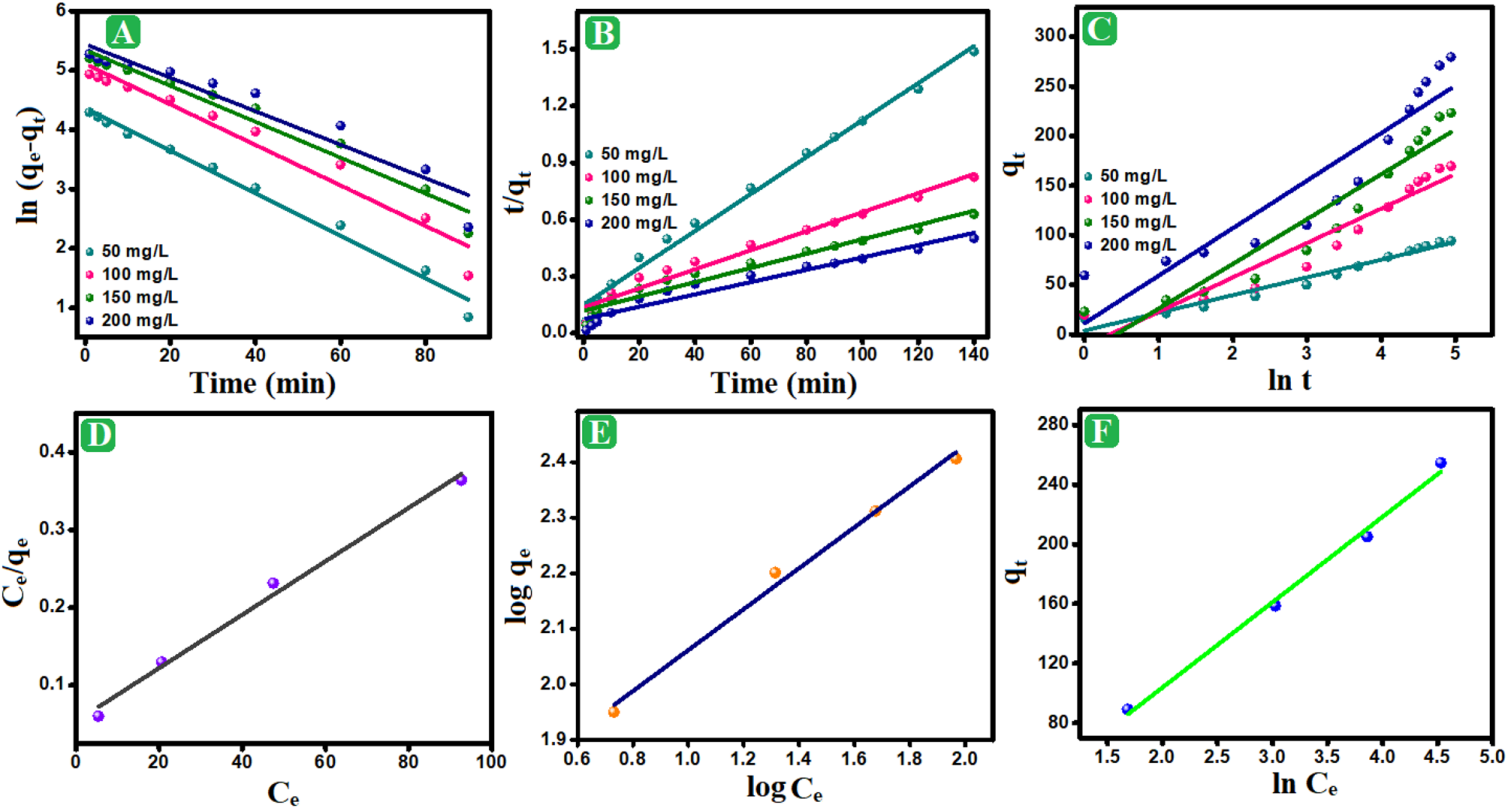
(**A**) Pseudo first order, (**B**) Pseudo second order, (**C**) Elovich, (**D**) Langmuir, (**E**) Freundlich, and (**F**) Temkin of the o-NP adsorption onto Fe_3_O_4_/AP-coke/N-Cs composite.

To recognize the responsible mechanism pathway for the o-NP adsorption onto Fe_3_O_4_/AP-coke/N-Cs composite, linear kinetic equations like Elovich, Pseudo first order, and Pseudo second order (Table S1) were applied in analyzing the experimental results^53,54^. Figure 6A,B indicate that the o-NP adsorption is best modeled by Pseudo second order since the R^2^ values of Pseudo second order at a wide scale of o-NP concentrations are higher than those of Pseudo first order and the SSE value of Pseudo second order are lower than of those of Pseudo first order, as elucidated in Table 1. Besides, the propinquity between the experimental equilibrium capacity of o-NP and the theoretical values that are derived from Pseudo second order is another clue to prove the suitability of Pseudo second order to represent the o-NP adsorption. This finding denoted the controlling of the chemosorption pathway on the o-NP adsorption onto Fe_3_O_4_/AP-coke/N-Cs composite. Interestingly, Elovich model Fig. 6C suggested the irreversibility of the o-NP adsorption onto Fe_3_O_4_/AP-coke/N-Cs composite, where the adsorption rate of o-NP molecules was quite higher than their desorption rate.

**Table 1:** Pseudo first order, pseudo second order, and Elovich models parameters for adsorption of o-NP ion onto Fe_3_O_4_/AP-coke/N-Cs composite.

Kinetic parameters	Concentration (mg/L)				
	50	100	150	200	
q_e,exp_. (mg/g)	89.20±2.36	158.73±3.42	205.07±5.68		254.60±6.62
Pseudo first order					
q_e,cal._ (mg/g)	88.90	208.57	282.47		327.15
k_1_ (min^–1^)	0.0174	0.0172	0.016		0.014
R^2^	0.948	0.952	0.957		0.918
SSE	0.163	0.520	0.309		0.625
Pseudo second order					
q_e,cal_ (mg/g)	102.56	198.80	264.55		307.69
k_2_ (g. mg^–1^. min^–1^)	0.0006	0.0002	0.00014		0.00012
R^2^	0.991	0.979	0.964		0.947
SSE	0.022	0.019	0.017		0.016
Elovich					
α (mg/g min)	22.30	24.48	29.85		62.83
β (g/mg)	0.056	0.029	0.022		0.020
R^2^	0.980	0.980	0.984		0.980
SSE	3.31	29.97	62.09		88.91

### Isotherm study

For the further realization of the dominant adsorption pathway on the adsorption of o-NP onto Fe_3_O_4_/AP-coke/N-Cs composite, the equilibrium data were inspected by the linear isotherm equations (Table S2). Figure 6D,E and Table 2 demonstrated that chemical and physical adsorption pathways worked together to adsorb o-NP onto the active species of Fe_3_O_4_/AP-coke/N-Cs composite since the R^2^ and SSE values of Langmuir, Freundlich, and Temkin are almost equal^55,56^. The Langmuir model defined that the maximal adsorption capacity of o-NP was 291.55 mg/g. Freundlich model assured the favorability of Fe_3_O_4_/AP-coke/N-Cs to adsorb o-NP molecules in which the n value exceeded two. The Temkin model (Fig. 6F) implied the controlling of physisorption since the b value was lower than 80 kJ/mol^57^.

**Table 2:** Parameters of Langmuir, Freundlich, and Temkin isotherms for the o-NP adsorption onto Fe_3_O_4_/AP-coke/N-Cs composite.

Isotherm model	Parameters	Value
Langmuir	q_max_ (mg/g)	291.55
	K_L_ (L/mg)	0.064
	R^2^	0.987
	SSE	4.35
Freundlich	n	2.72
	K_F_ ((mg/g) (L/mg)^1/n^)	39.81
	R^2^	0.989
	SSE	4.08
Temkin	k_T_ (L/mg)	0.832
	b_T_ (kJ/mol)	0.433
	R^2^	0.990
	SSE	3.88

### The plausible adsorption mechanism

**Figure 7:**
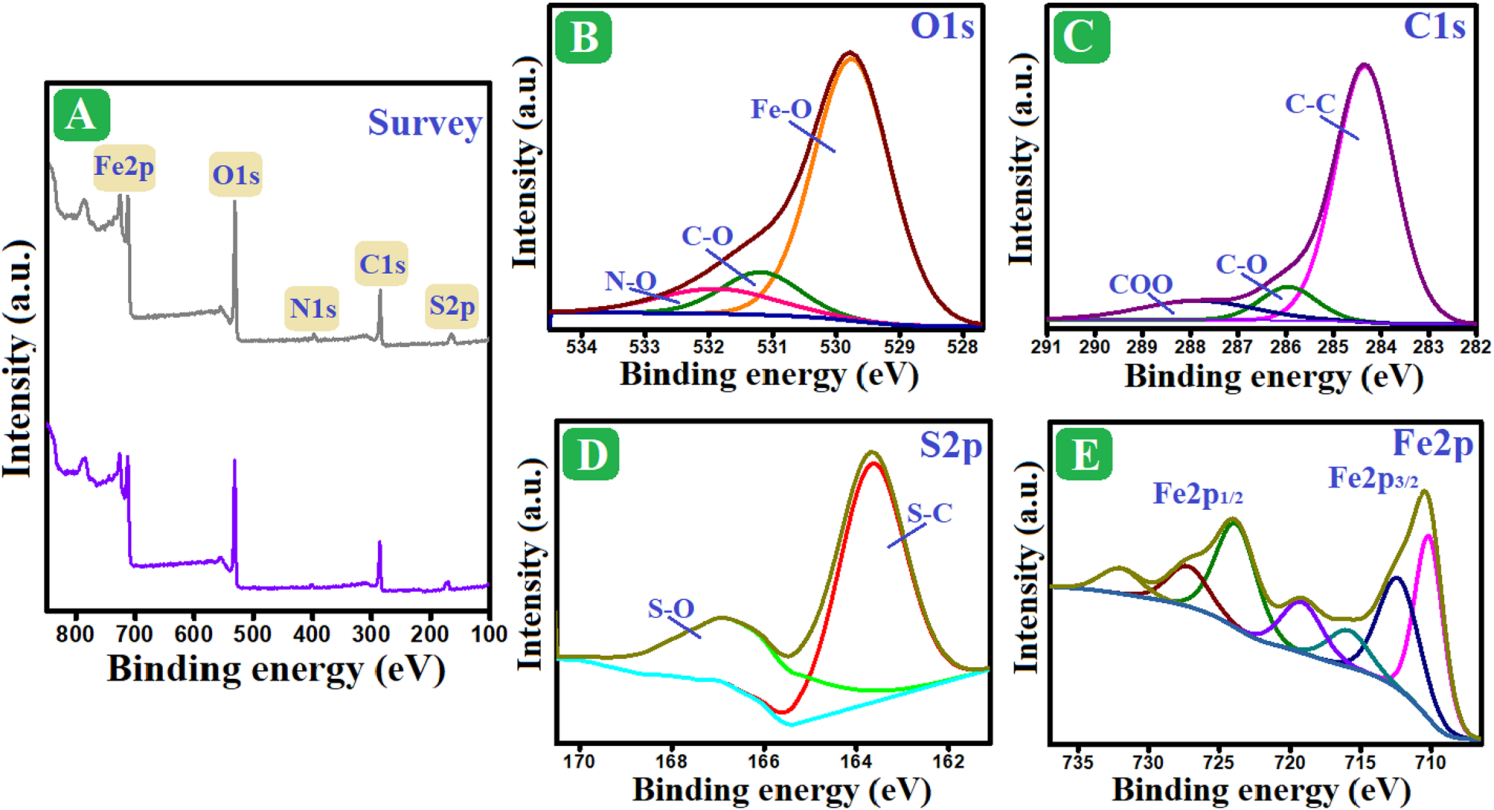
(**A**) Wide spectrum of Fe_3_O_4_/AP-coke/N-Cs before/after the o-NP adsorption, and (**B**) O1s, (**C**) C1s, (**D**) S2p, and (**E**) Fe2p of used Fe_3_O_4_/AP-coke/N-Cs composite.

The FTIR spectrum of the o-NP-loaded Fe_3_O_4_/AP-coke/N-Cs composite (Fig. S4) revealed new bands at 1407 and 1543 cm^-1^, which correspond to NO_2_ stretching^58^. In addition, the observed band at 867 cm^–1^ attributes to the benzene ring^59^. Furthermore, by comparing the wide-scan spectra of Fe_3_O_4_/AP-coke/N-Cs before and after the o-NP adsorption (Fig. 7A), it was noticed an increase in the intensity of nitrogen peak. These findings evinced the occurrence of the o-NP adsorption onto the Fe_3_O_4_/AP-coke/N-Cs surface. In addition, the oxygen spectrum of used Fe_3_O_4_/AP-coke/N-Cs signified the belonging peak to N-O which is another clue for evincing the adsorption of o-NP onto the composite (Fig. 7B). Consequently, it was quintessential to understand how the o-NP molecules attach to the adsorption species of the Fe_3_O_4_/AP-coke/N-Cs composite. The o-NP molecules are in the molecular form until pH = 7.23, while they convert to anions species when pH increases over 7.23. Therefore, the electrostatic interaction could not contribute to the adsorption of o-NP in acidic and neutral media. Electrostatic repulsion negatively impacts the aptitude of the anionic o-NP molecules onto the negatively charged binding species of Fe_3_O_4_/AP-coke/N-Cs in the alkaline medium.

The H-bonds could be formed between the nitrogen and oxygen of o-NP molecules and the plentiful hydrogen onto the Fe_3_O_4_/AP-coke/N-Cs surface, and vice versa. Notably, nonyl-functionalization strengthens the H-bonds between Fe_3_O_4_/AP-coke/N-Cs and o-NP molecules since it provides abundant hydrogen atoms onto the composite surface. In addition, the presence of a nonyl group onto Fe_3_O_4_/AP-coke/N-Cs could enhance the hydrophobic interactions between Fe_3_O_4_/AP-coke/N-Cs and o-NP molecules. The π-π interaction is an influence mechanism pathway that could participate in the o-NP adsorption onto Fe_3_O_4_/AP-coke/N-Cs via their aromatic rings. H-bonding, π-π interaction, and hydrophobic interactions were confirmed by the peaks shifting of the carbon spectrum of the used Fe_3_O_4_/AP-coke/N-Cs composite (Fig. 7C).

The electron donner/acceptor interactions between (i) electron donner groups of Fe_3_O_4_/AP-coke/N-Cs; sulfonic, hydroxyl, and benzene ring and nitro group of o-NP (electron acceptor group), and (ii) the electron acceptor groups of Fe_3_O_4_/AP-coke/N-Cs (carboxyl) and electron donner groups of o-NP; hydroxyl and benzene ring. Moreover, the iron species of the composite could attach to o-NP molecules by forming coordination bonds. These suggestions were inferred by the peaks shifting of the sulfur, oxygen, iron, and nitrogen spectra after the o-NP adsorption (Figs. 7D,E and S3).

In one word, several mechanism pathways could contribute to the adsorption process of o-NP onto Fe_3_O_4_/AP-coke/N-Cs; (i) chemical pathways such as H-bonding, π-π interaction, electron donner/acceptor interaction and coordination bonds, and (ii) physical pathways like hydrophobic interaction. These suggestions are consistent with kinetic and isotherm results that implied the participation of chemical and physical pathways in the o-NP adsorption.

### Comparison with other adsorbents

A comparison study was executed between the fabricated magnetic composite with other reported adsorbents was presented in Table 3. Remarkably, the developed Fe_3_O_4_/AP-coke/N-Cs composite exposed ultimate adsorption aptitude towards o-NP compared to other adsorbents. From the comparison study, the developed Fe_3_O_4_/AP-coke/N-Cs composite recorded the highest adsorption capacity value of 291.55 mg/g which was accomplished in a shortest equilibrium time (100min). These results suggest the potential applicability for adsorptive removal of o-NP.

**Table 3:** Comparison of maximum adsorption capacity and equilibrium contact time of Fe_3_O_4_/AP-coke/N-Cs composite with other adsorbents for the o-NP removal.

Adsorbent	q (mg/g)	Eq. time (min)	Ref.
Biochar from Hizikia fusiformis	10.39	360	^60^
Activated carbon prepared from winery wastes	179	300	^61^
Nano zeolite (NZ)	125.7	150	^62^
NiAl-layered double hydroxide	77.10	100	^63^
Hyacinth-activated carbon	47.62	180	^64^
Clinoptilolite	60	180	^65^
Fe_3_O_4_/AP-coke/N-Cs composite	291.55	100	This study

### Removal of o-NP from actual wastewater

Actual wastewater samples were collected from the industrial drain of a pigment factory in Alexandria, Egypt. The specifications of the collected actual wastewater before and after the treatment process are listed in Table 4. The o-NP concentration in the actual wastewater sample was about 57.23 mg/L. Surprisingly, the removal efficacy of the o-NP in the actual wastewater by Fe_3_O_4_/AP-coke/N-Cs was 82.45 % after 100 min, clarifying the high efficiency and applicability of the as-fabricated composite in the actual wastewater remediation.

**Table 4:** The specification of the actual wastewater before and after treatment.

Parameters	Specifications (before treatment)	Specifications (after treatment)
pH	9.4	7.5
Turbidity (NTU)	355	310
Total dissolved solvents (ppm)	626	531
Conductivity (µc/cm)	1100	974

### Regeneration study

**Figure 8:**
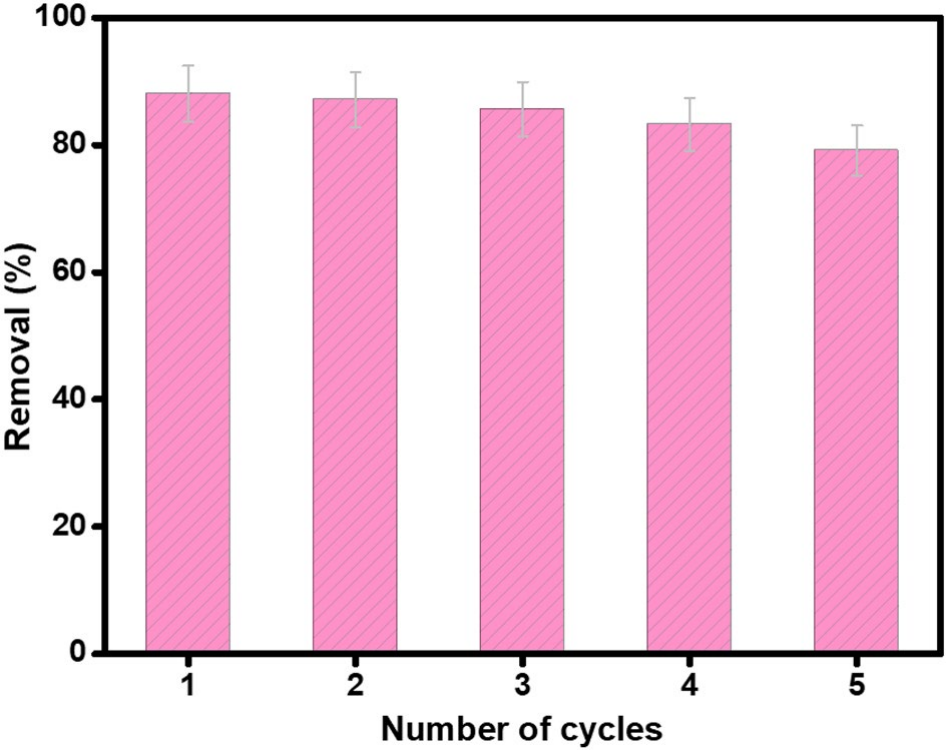
Regeneration study of Fe_3_O_4_/AP-coke/N-Cs for five o-NP adsorption/desorption cycles.

Recycling test was performed to evince the regeneration ability of the as-synthesized Fe_3_O_4_/AP-coke/N-Cs magnetic composite. The test of regeneration proceeded by adding 0.01 g of Fe_3_O_4_/AP-coke/N-Cs in 20 mL of o-NP and the adsorption process was kept under stirring for 100 min. Then, the Fe_3_O_4_/AP-coke/N-Cs composite was collected and washed by 1 M NaOH followed by distilled water. This former procedure was repeated for five o-NP adsorption/desorption cycles. Surprisingly, the recycling test results (Fig. 8) implied that the removal (%) of o-NP declined by 8.93 % after the 5th cycle, reflecting the excellent regeneration ability of Fe_3_O_4_/AP-coke/N-Cs.

## Conclusion

This study reported the formulation of Fe_3_O_4_/AP-coke/N-Cs magnetic composite for adsorptive removal of o-NP. Factors affecting the adsorption process were investigated through a series of batch adsorption studies. High adsorption performance towards o-NP was accomplished by Fe_3_O_4_/AP-coke/N-Cs composite at pH 6 compared to its individual components. A sequence of adsorption isotherm and kinetic studies concluded that the adsorption of o-NP onto the composite surface was fitted to Langmuir, Freundlich, and Temkin isotherm models with a maximum adsorption capacity of 291.55 mg/g at 25 ºC and followed the pseudo second order kinetic model. Moreover, the gained XPS results assumed that the adsorption mechanism comprises H-bonding, π-π interaction, hydrophobic interactions and electron donor-acceptor interactions. Overall, the proposed magnetic composite's outstanding adsorption performance, better reusability and simple separation characteristic point to its potential application for the adsorptive removal of phenolic contaminants from aquatic systems.

### Supplementary Information

Supplementary Information.

## Data Availability

The data sets used and analyzed during the current study are available from the corresponding author on reasonable request.
