# Supplementary material for: Sustainable synthesis of magnetic petroleum coke/nonanyl chitosan composite for efficient removal of o-nitrophenol
Source: Sci Rep. 2024 Jun 24;14:14463. doi: 10.1038/s41598-024-64117-1 (PMC11196280; doi:10.1038/s41598-024-64117-1)

**Sustainable synthesis of magnetic petroleum coke/nonanyl chitosan composite for efficient removal of o-nitrophenol**

Ahmed M. Omer ^1,*,^ Abdelazeem S. Eltaweil ^2, 3^, Aly M. Abdelhamed ^2, 4^, Eman M. Abd El-Monaem ^2^,

, Gehan M. El-Subruiti ^2^

^1^ Polymer Materials Research Department, Advanced Technology and New Materials Research Institute (ATNMRI), City of Scientific Research and Technological Applications (SRTA-City), New Borg El-Arab City, P. O. Box: 21934, Alexandria, Egypt

^2^ Department of Engineering, Faculty of Technology and Engineering, University of Technology and Applied Sciences, Sultanate of Oman

**^3^** Chemistry Department, Faculty of Science, Alexandria University, Alexandria, Egypt

^4^ Environmental department, EPROM-MIDOR Refinery, P. O. Box: 1001, Alexandria, Egypt

*Correspondence: Ahmed M. Omer ([ahmedomer_81@yahoo.com](mailto:ahmedomer_81@yahoo.com))

**Figure S1.** Zeta potential of Fe_3_O_4_/AP-coke/ N-Cs composite at different pH media.


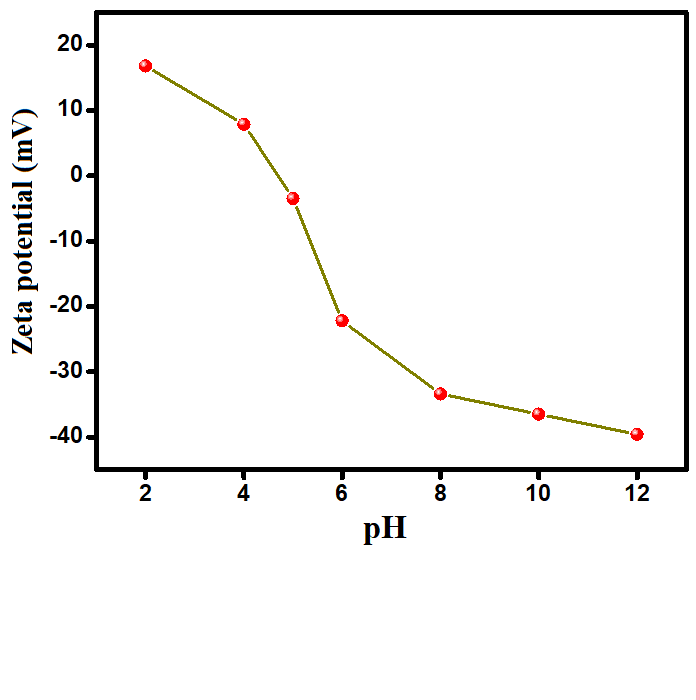


**Figure S2.** Nitrogen spectrum of Fe_3_O_4_/AP-coke/ N-Cs composite.


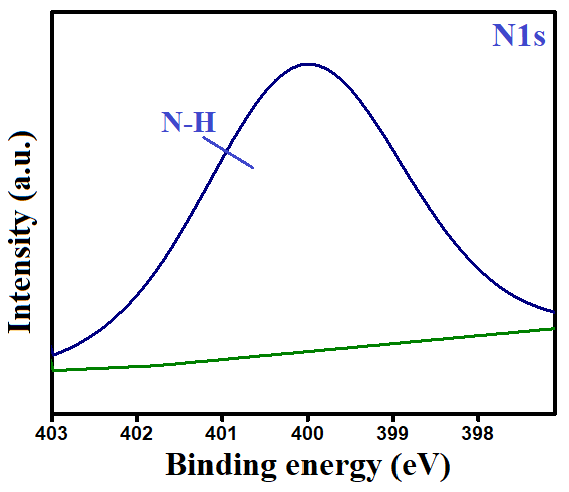


**Figure S3.** Nitrogen spectrum of the used Fe_3_O_4_/AP-coke/ N-Cs composite.


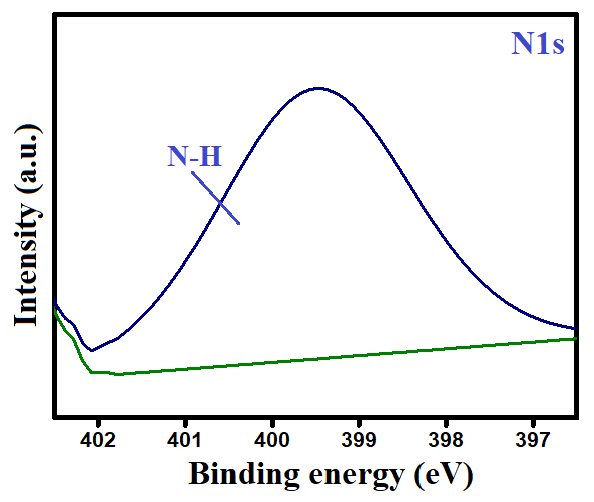


**Table S1.** Non-linear equations of the applied adsorption kinetic models

| **Kinetic Model** | **Equation** |
| --- | --- |
| **Pseudo 1^st^ order** | $\ln(q_{e}-q_{t})=\ln q_{e}-k_{1}\left( t \right)$**(1)** |
| **Pseudo 2^nd^ order** | $t/{q_{t}}=1/{k_{2}q_{e}^{2}+ 1/{q_{e}}\left( t \right)}$ **(2)** |
| **Elovich** | $q_{t}=\frac{1}{\beta}\ln\left( \alpha\beta\right)+ \frac{1}{\beta}$ ln (t)  **(3)** |

Where, q_t_ and q_e_ are amounts of o-NP uptakes at time t and equilibrium, respectively. k_1_ and k_2_ are the rate constants of Pseudo 1^st^ order and Pseudo 2^nd^ order, respectively. Furthermore, α and β are Elovich coefficients that represent the initial adsorption rate and the desorption coefficient, respectively, also related to the extent of surface coverage and activation energy for chemisorption.

**Table S2.** Non-linear equations of the applied adsorption isotherm models

| **Model** | **Equation** |
| --- | --- |
| **Langmuir** | $\frac{C_{e}}{q_{e}}=\frac{1}{K_{L} q_{m}}+\frac{C_{e}}{q_{m}}$ **(4)** |
| **Freundlich** | $\log q_{e}=\log K_{F}+\frac{1}{n}\log C_{e}$ **(5)** |
| **Temkin** | $q_{e}=B lnA+B\ln C_{e}$ **(6)** |

Where, q_m_ and K_L_ are the maximum monolayer adsorption capacity and Langmuir constant, respectively. n and K_F_ are Freundlich constants. A is the equilibrium binding constant and $B=\frac{\mathrm{RT}}{b}$ , b is Temkin constant related to heat of adsorption.

**Figure S4.** FTIR of Fe_3_O_4_/AP-coke/N-Cs before/after the o-NP adsorption.


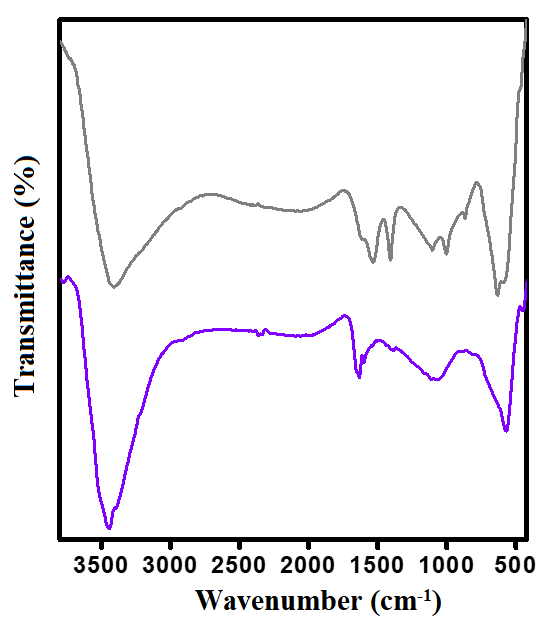

Supplement: Supplementary file 1 — Supplementary Information. [file 41598_2024_64117_MOESM1_ESM.docx]
